# Sensitisation to Cor a 14 and Cor a 9 is a risk marker for severe hazelnut allergy in children

**DOI:** 10.1186/2045-7022-4-S2-O15

**Published:** 2014-03-17

**Authors:** Lorna Garnier, Clémence Massip, Sébastien Viel, Jacques Bienvenu, Françoise Bienvenu

**Affiliations:** 1 Lyon University Hospital, Immunology Laboratory, Pierre-Bénite, France

## Background

Hazelnut-allergic children express variable clinical phenotypes. Tests for the diagnosis of hazelnut allergy are usually conducted on children presenting clinical symptoms following hazelnut ingestion, but also as screening tests after the discovery of a food allergy, mainly to tree nut or peanut. It is important to determine if molecular allergens could be markers of severity in hazelnut allergy.

## Objectives

To evaluate the usefulness of hazelnut molecular allergens in children with suspected hazelnut allergy. To study whether these allergens might predict the severity of hazelnut allergy.

## Method

41 hazelnut-sensitized children (69% males, mean age: 8.1 years) were divided into 2 groups: - 29 patients with severe allergy i.e. angioedema, urticaria, emesis or hoarseness after ingestion of crude or roasted hazelnut, or children with positive prick-tests to roasted hazelnut. - 12 patients with mild allergy i.e. an oral allergy syndrome after ingestion of crude hazelnut, or children under hazelnut free diet due to a strongly positive prick-test to crude hazelnut performed after an allergic reaction to another tree nut or peanut. Specific IgE against 4 hazelnut allergens (rCor a 1, rCor a 8, rCor a 9, rCor a 14) were determined for each patient, using the ImmunoCap® 250 system (Thermo Fisher).

## Results

On the whole population, IgE positivity to Cor a 14 (73%), Cor a 9 (56%) and Cor a 1 (37%) are the most frequent. Conversely, only 2 children had positive IgE against Cor a 8. Children with severe allergy were all positive for Cor a 14 (93%) or Cor a 9 (76%), and 69% were positive for both. Only 10% of them had IgE against Cor a 1. Children with mild allergy were all IgE positive for Cor a 1. 67% were positive only for Cor a 1. 25% were also positive for Cor a 14 and 8% for Cor a 9. None of them were positive for both Cor a 9 and Cor a 14. Most of these children were pollinic (75%).

## Conclusion

2 clinical phenotypes of hazelnut allergy can be clearly distinguished using molecular allergens: - Sensitization to seed storage proteins (Cor a 9 or Cor a 14) is a strong marker of severe allergy, even more specific when both molecular allergens are positive. - Sensitization to Cor a 1 defines a PR-10 profile specific for children with a mild allergy which is often associated with pollen allergy. Thus, testing Cor a 9, Cor a 14 and Cor a 1 specific IgE is of great help in identifying children at risk for severe allergic reactions to hazelnut.

